# Evaluation of the eOSCE for testing clinical skills

**DOI:** 10.3389/fvets.2023.1196311

**Published:** 2023-08-17

**Authors:** Svenja Berendes, Elisabeth Schaper, Andrea Tipold, Sandra Wissing

**Affiliations:** ^1^Centre for E-Learning, Didactics and Educational Research (ZELDA), Clinical Skills Lab, University of Veterinary Medicine Hannover, Foundation, Hannover, Germany; ^2^Centre for E-Learning, Didactics and Educational Research (ZELDA), E-Learning Consulting, University of Veterinary Medicine Hannover, Foundation, Hannover, Germany; ^3^Clinic for Small Animals, Neurology, University of Veterinary Medicine Hannover, Foundation, Hannover, Germany

**Keywords:** eOSCE, E-assessment, clinical skills, clinical assessment, veterinary education, formative test, questionnaire based study

## Abstract

The OSCE is a proven instrument for testing practical skills of students, not only in the (veterinary) medical field. At the University of Veterinary Medicine Hannover, Foundation (TiHo), students must complete a 10-week rotation in one of the university’s own clinics during the practical year in the 9th and 10th semesters in addition to other practical training. Within this framework, the first training for acquiring clinical practical skills with a formative examination (OSCE; Objective Structured Clinical Examination) was developed in the Clinical Skills Lab (CSL) in 2014. Since 2018, the TiHo has been the first veterinary medical training institution in Germany to conduct electronic OSCEs (eOSCEs). In the future, eOSCEs will also be introduced as a summative examination at the TiHo. For this reason, students in the practical year of the winter semester 2021/2022 and summer semester 2022 as well as former and current examiners were surveyed regarding their satisfaction with the eOSCE examination format as part of this study. The 175 analyzed questionnaires from students and 27 analyzed questionnaires from examiners showed a high level of satisfaction with the eOSCE overall, but at the same time indicated problems that should be ironed out before it is introduced as a summative examination in the state examination. In addition to its standardization and objectivity, the examination format also convinced with its quality-assurance review procedure and the direct feedback possibility with regard to the students’ performance.

## 1. Introduction

The Ordinance on the Approbation of Veterinarians (TAppV) defines the structure of veterinary medicine studies for all German educational institutions (1). At the German educational institutions, veterinary medical training consists of a scientific-theoretical part and a practical part. At the University of Veterinary Medicine Hannover, Foundation (TiHo), the practical year (PY) takes place in the 9th and 10th semesters with the aim of integrating students better into scientific and clinical-practical activities. Small group teaching at the university’s clinics is intended to improve students’ practical training with patients, while at the same time, an “orientation phase” gives students the opportunity to set species-specific priorities (2). In addition to practical training in various veterinary departments, the PY at the TiHo includes a minimum 10-week rotation in a university-owned veterinary clinic or paraclinic selected by the students. Within this rotation, a list of duties drawn up by the clinic or institute including relevant veterinary skills has to be worked through (3).

### 1.1. Introducing skills-lab-training at the TiHo

“Clinical competence includes the ability to communicate, take a medical history, and perform manual and instrumental examinations” (4) as well as planning and interpreting diagnostics, therapy and patient education (5). The European Coordination Committee for Veterinary Training (ECCVT) has compiled a list of “Day One Competences” based on EU directives and regulations and several publications, including those of the World Organization for Animal Health (OIE) and the European Association of Establishments for Veterinary Education (EAEVE). These competencies are defined as minimum standards required for professional development after graduation. Graduates with “Day One Competences” should be able to, and also have the confidence to independently put the fundamentals of veterinary medicine into practice. At the same time, they should be able to assess when they need to seek the advice or guidance of an experienced colleague (6). The OIE defines competencies as a combination of cognitive and affective skills, practical skills, and aptitude (7), while the EAEVE, in collaboration with the Federation of Veterinarians of Europe (FVE) and Animal WelfAre Research in an Enlarged Europe (AWARE), emphasized the importance of “Day One Competencies” in animal welfare in their report (8). The Royal College of Veterinary Surgeons (RCVS), United Kingdom, has produced a model of “Day One Competences” outlining the different areas in which veterinary surgeons must prove their competency after graduation. In addition to veterinary skills, these include competencies in personality, business and administration, and communication (9).

In the CSL, students are taught relevant clinical-practical activities that are important for their later everyday work as veterinarians as so-called first-day skills. These include, for example, dressing, suturing and injection techniques, or the placement of a peripheral venous catheter. Hand grips and skills are practiced on models and simulators. In this way, students learn, practice, and repeat skills in a protected environment prior to surgery on live animals. This helps students to be able to perform the hand grips more routinely and to use them more confidently on a live animal, which is ultimately important for animal welfare and protection.

In 2014, in cooperation with the Clinic for Small Animals at the TiHo, the development and implementation of a one-week teaching intervention (skills lab training) in the CSL for acquiring clinical-practical skills for PY students took place. The learning objectives defined in a blueprint were assigned to the thematic blocks of communication, ward/clinic surgery, anesthesia, and imaging. The success of the training was assessed by means of an objective structured clinical examination (OSCE) (10).

The OSCE examination format was first introduced by Ronald Harden in 1975 and has since been used successfully in many university departments (11). An OSCE consists of a course comprising several stations, which you go to in rotation. Within a specified testing time, the student must complete a predetermined task at each of these stations. At the beginning of each station, the student receives precise written instructions (“scenario”) on what he/she must perform at that station. At the end of the examination time, the examination is terminated and the student moves to the next station within a changeover time. An examiner is at each station, observes the student’s performance, and evaluates the performance against a pre-defined objective-structured checklist. This approach allows multiple students to complete an OSCE course simultaneously (12). While traditional (written) forms of examinations primarily assess students’ theoretical knowledge, the OSCE enables performance-based performance review (13). Since introducing the Skills Lab training with OSCE for PY students at the Clinic for Small Animals in 2014, the concept has been extended to other TiHo clinics. Currently, trainings with an eOSCE for PYs at the Clinic for Small Animals (since 2014), Clinic for Small Ruminants (since 2015), Clinic for Horses (since 2020), and the Clinic for Small Mammals, Reptiles, and Birds (since 2021) take place in the CSL, each before or at the beginning of the clinic rotation. Participation in the skills lab training and OSCE is mandatory. The eOSCE is conducted as a formative examination and serves as a support for the students’ learning process as well as a self-examination and feedback on their current learning progress. In the context, feedback to students includes a relevant function to reveal existing deficits and the opportunity to improve their own performance (14, 15). In the subsequent clinic rotation, students can set priorities based on their OSCE result and work on deficits under professional guidance.

Since 2018, the TiHo has been the first veterinary medical training institution in Germany to conduct the OSCE in electronic form as eOSCE. The entire exam preparation is done in digital form in cooperation with an external service provider. The examiners process the checklists on tablets and the results of the eOSCE can be downloaded and transmitted to the students directly after the examination, as there is no need for manual evaluation of the checklists. The reasons for switching from a paper-based to an electronic format were to conserve material and human resources and to save time by evaluating the checklists electronically. As part of a current dissertation study at CSL, both formats are being compared and the eOSCE evaluated.

## 2. Materials and methods

The content of the skills lab training and eOSCEs in the CSL is directly related to the teaching of the veterinary clinics of the TiHo. Thus, the development of the training concept as well as the preparation of the teaching materials was done in close cooperation with the respective lecturers of the clinics.

At the beginning of the development, learning objectives and clinic-specific and clinic-relevant focal points were defined, and subsequently, a schedule for the skills lab training was designed. All training content was developed and evaluated based on current veterinary teaching standards.

Once the training has been developed, clinically relevant examination stations were created based on the training concept for the eOSCE. This was done using objective structured checklists for each exam station, which were outlined different aspects of the action to be examined to the examiners (16).

### 2.1. eOSCE checklist structure

An objective-structured eOSCE checklist at the CSL was created in collaboration with several CSL staff members and the lecturers of the respective veterinary clinic. Each checklist was based on the technical content of the respective training and followed a cross-clinic standardized structure consisting of the headings “Clinical Skills,” “Overall Impression,” “Feedback,” and “Tips.”

**Figure 1:**
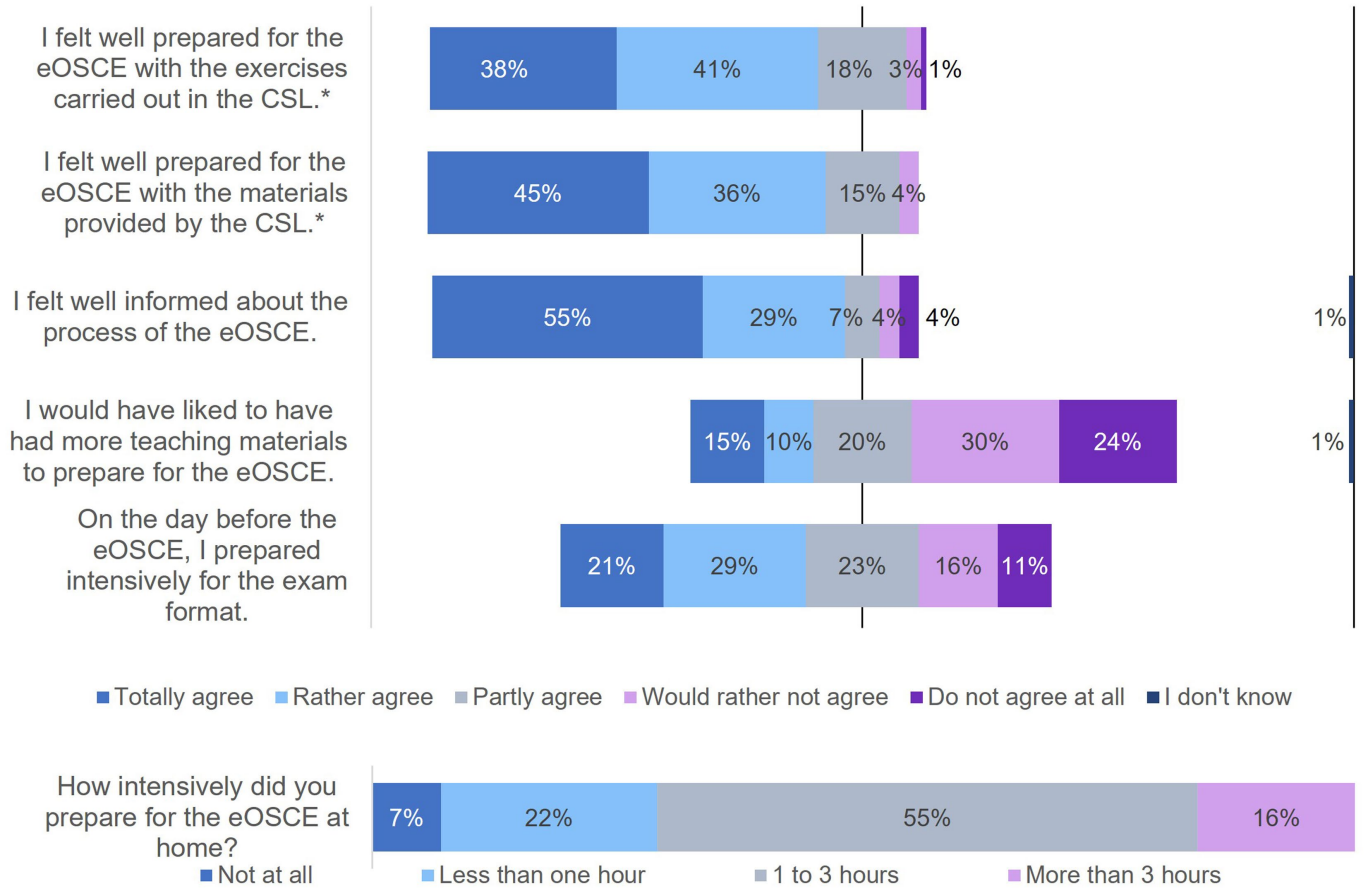
Evaluation of the eOSCE by students of the University of Veterinary Medicine Hannover in the period from September 2021 to July 2022: preparation for eOSCE (*n* = 175). * = Due to correct rounding, the total is not exactly 100%.

Items under “Clinical Skills” followed the chronological sequence of the skill being tested (see Figure 1). This facilitated the examiner’s work, avoided unnecessary scrolling on the tablet, and ensured that the student received the necessary attention at the station to assess his/her performance. A multi-stage review process ensured that the items were formulated in such a clear and comprehensible way that examiners are left with no room for interpretation for a subjective evaluation. This had the advantage that anyone with a (veterinary) medical background could be appointed as an examiner for the eOSCE and made it possible to compensate for any staff shortages.

Furthermore, the items under “Clinical Skills” differentiated between special items that are specific to the practical skill at the respective station and general items that are relevant for all stations. Special items included, for example, wrapping the paw with a padding bandage at the station paw dressing, while “Adherence to an aseptic work method” fell under general items.

“Overall impression,” “Feedback,” and “Tips” were sent to students with their eOSCE score and were not included in the percentage rating of performance in the eOSCE station.

### 2.2. Review process

Every eOSCE checklist which was compiled by the CSL members of staff followed a standardized structure and was first reviewed by employees of the respective clinic and, if necessary, professional comments and requests for changes are added. The responsible CSL employee incorporated the corrections into the checklist and then added them to the specific TiHo OSCE examination platform.

Subsequently, each checklist went through a special quality assurance review process directly in the exam platform. The process began with a formal review by trained employees from the e-learning Consulting department. The focus here was on didactic aspects. The items on the checklist were checked for clarity and comprehensibility. If no formal changes were required, the checklist was released directly for the technical review and checked for technical content by the respective clinic.

**Figure 2:**
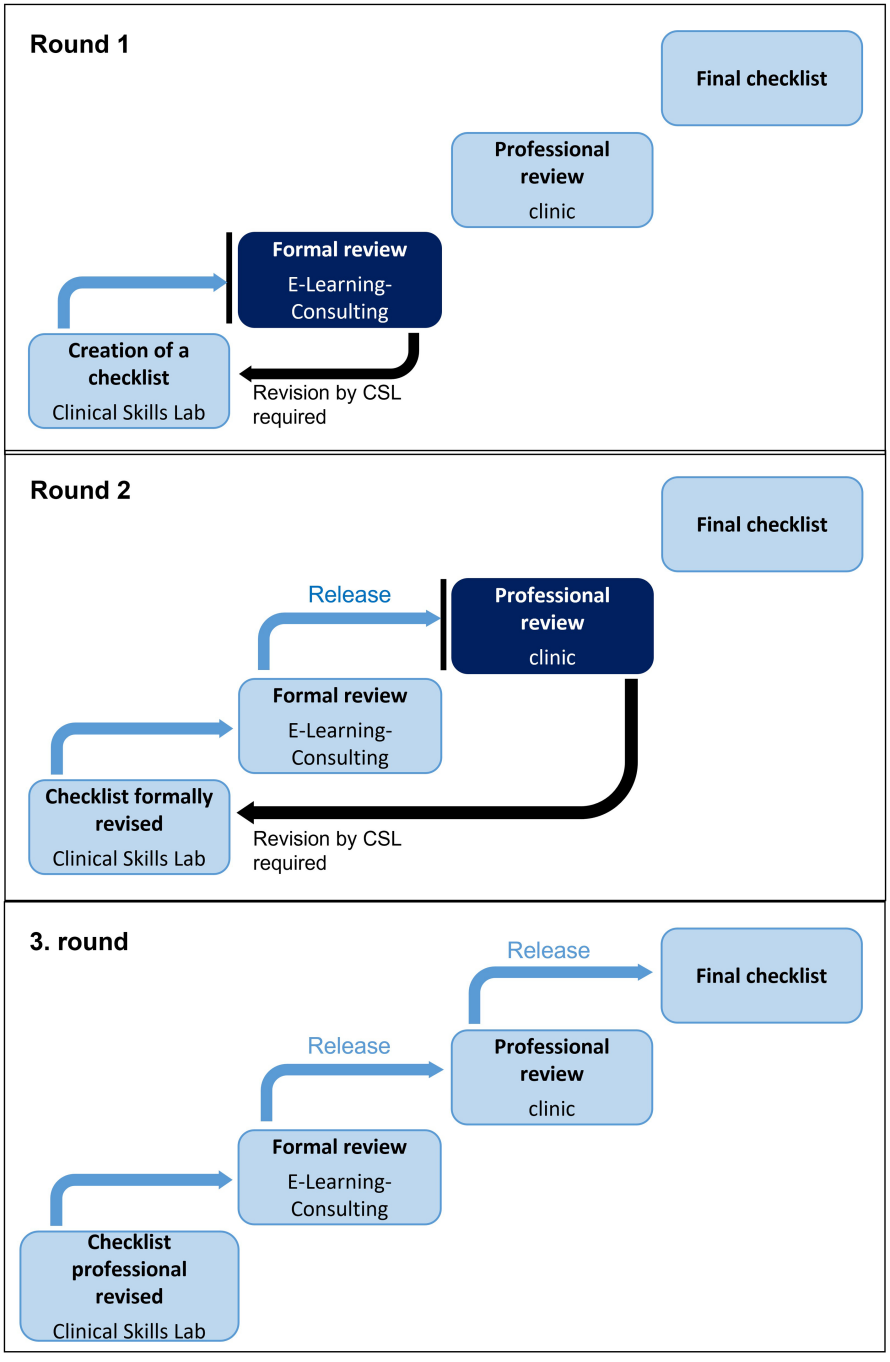
Flow diagram of the review process at the CSL at the University of Veterinary Medicine Hannover for necessary formal and technical corrections.

In the event that a checklist was not accepted in the formal review and changes were necessary, the system sent a message to the CSL staff member(s) to make the revisions in a timely manner (see Figure 2). The checklist was then routed again to the formal review. If no further changes were necessary, the formal review was successfully completed and the technical reviewers, veterinarians of the respective clinic, were informed by the system to perform the technical review. The focus here was on content-related aspects specific to the animal species and clinic. If a checklist did not pass the technical review, a message was sent to the CSL employee. This person made the appropriate changes in the system and then sent the checklist back to the formal review. The checklist then had to be formally reviewed again and, after a successful review, was released for the technical review. This cycle of formal and technical review and corresponding revisions by the CSL employee was repeated until the checklist had successfully passed both reviews.

### 2.3. Evaluation system of an eOSCE checklist

Traditionally, checklists with binary items are used in an OSCE to record the correct performance of sub-steps of the examination task (17).

Also, the TiHo eOSCE checklists usually consisted of binary (e.g., fulfilled, not fulfilled) items and, in exceptional cases, of non-binary items. For the non-binary items, students had the opportunity to score partial points for a skill in the exam (partially fulfilled). The items on the checklist for which partial points could be earned were determined by an internal CSL scoring procedure.

Items were scored according to a set point system, ascending from “less important” (1 point), to “important” (2 points), to “very important” (3 points). An example of a “less important” item in a suturing technique checklist is the correct handling of the needle holders and scissors in the thumb/ring finger grip. Placing knots adjacent to the wound gap represents an “important” item, while adherence to an aseptic technique represents an example of a “very important” item. Sub-steps that were difficult to implement were assigned an extra point for a “high degree of difficulty.” This included, for example, the correct surgical filament for a suture or the adaptation of the wound edges over the entire length of the wound gap.

The items in new checklists were evaluated internally by CSL employees. In the first step, each employee evaluated the items in the checklist separately. Then, in a joint evaluation process, the scoring was discussed and a final score was determined. The individual points for the items resulted in a total score (modified according to Angoff). The total score for the various eOSCE stations varied depending on the weighting of the individual stations.

In the case of the overall impression, the examiner evaluated the student using a global rating consisting of the points “insufficient,” “poor,” “satisfactory,” “good,” and “very good.” The overall impression was communicated to the student following the eOSCE during the feedback meeting.

The review and evaluation process standardizes the checklists in the CSL. However, with each newly designed training for additional clinics, existing checklists with cross-clinic skills such as suturing techniques are also subjected to a further review process and undergo an ongoing editing process.

### 2.4. Exam compilation

The number of examination stations of 14 to 18 required in the literature as well as the estimated time per station of 10–15 min each for the performance of a reliable OSCE (18, 19) is currently not used at the TiHo due to the formative examination character of the eOSCEs, as this would result in a considerable expenditure of time and personnel. Whether the required reliability of 0.8 (18, 19) can also be achieved with a shorter examination time and fewer examination stations in the eOSCE in the CSL was currently being investigated as part of a thesis.

The stations to be examined in an eOSCE were determined jointly by CSL staff and that of the respective veterinary clinic based on a blueprint. Depending on the number of students, eOSCEs were conducted at the TiHo with a maximum of 10 stations supervised by examiners. If more than 10 students participated in an eOSCE, there were additional unsupervised stations where students complete multiple-choice tests or ECG evaluations, for example.

### 2.5. Professional and technical training of the examiners

The supervised exam stations in the eOSCE were staffed by pre-trained veterinarians or CSL student assistants and veterinarians from the respective veterinary clinic. To ensure the 30-min checklist preparation time required by the literature for examiners (20), checklists were provided to all examiners approximately 1 week prior to the eOSCE. In addition, there was an “Examiner Sheet” for each examination station, which explained the exact procedure of the station and pointed out possible special features with regard to the simulators used at the respective station. All examiners received previous technical training for the station to be examined, which included both theoretical background knowledge and successful performance of the clinical-practical skill on the simulator. Examiners who had no previous experience with the eOSCE at the TiHo additionally received technical training in the use of the tablet on the day of the examination by employees of the service provider.

### 2.6. eOSCEs procedure at the TiHo

The entire preparation for the eOSCEs took place in the eOSCE exam platform. An examination could only take place once the checklists for the stations had successfully passed both the formal and the technical review. This ensured the consistent quality of each eOSCE exam.

On the platform, the CSL staff member could enter all relevant data, such as the room schedule and the exam and changeover time for the eOSCE into the system. The data of the participating students were also stored in the system. Once all persons had been entered, the service provider created the eOSCE on this basis and automatically assigned the participants to their respective starting station.

One week before the scheduled examination date, all data had to be stored in the system and the examination had to be submitted to the service provider. The service provider checked the test with the help of an internal test run and eliminated any sources of error.

One day before the exam date, the technical set-up (e.g., set-up of WLAN connections for the examiners’ tablets) of the eOSCE was carried out by the service provider. The individual eOSCE stations were set up by CSL employees after the Skills Lab training. This ensured that students did not know the exam stations beforehand.

On the day of the exam, a team from the service provider was on site throughout the exam period to deal promptly with any technical problems. Technical equipment including tablets for the exam were provided by the service provider.

The service provider announced the start and end of the examination period with an acoustic signal. At the start of the examination, the checklist for the student(s) currently being examined opened automatically on the tablet. This prevented accidental opening of another student’s checklist. The checklist with the items was displayed to the examiners on their tablet. The students had to complete the respective station within a specified examination time. This time also represented the processing time in the checklist for the examiners. At the end of this period, the examiners had a time window for post-processing the checklist. In order to prevent individual items from being overlooked and thus not evaluated, items for which no evaluation had yet been carried out after the end of the examination period were highlighted in color. At the end of the post-processing time, the checklist was automatically closed and the next acoustic signal opened the checklist of the next student(s). Closed checklists could not be opened or changed manually afterwards.

The usability of the tablet was very intuitive and simple. If problems occured during the examination, employees from the service provider who were on site could react immediately and provide a replacement tablet if necessary. Entries already made by the examiners in the checklist were stored on an external server of the service provider and could be retrieved at any time.

Each exam station in the CSL was signposted with the respective station name; in the case of non-adjacent examination rooms, additional directional signs were installed. Students had 7 minutes per station to complete the examination, followed by a three-minute changeover period during which students moved to the next station. The follow-up checklist time for examiners, which was within the changeover time, was two and a half minutes. Depending on the veterinary clinic, a single eOSCE run took 67 (seven stations) to 97 min (10 stations). There was a maximum of four eOSCE runs per day.

After the eOSCE exam, all examiners gathered and gave a short feedback on the course of their station. The focus was primarily on any errors that students had made. Critical questions were asked as to whether there were any ambiguities or misunderstandings in the teaching unit preparing for the eOSCE. Following the examiner meeting, students in the group received general verbal feedback from the examiners and, in turn, were also allowed to provide their own feedback on the CSL training as well as the eOSCE. Once the exam results were available, students received written feedback on their own performance, consisting of the percentage achieved in the “Clinical Skills,” the “Overall Impression,” the “Feedback” and the “Notes” for each station.

### 2.7. eOSCE analysis

The eOSCE is currently used as a formative exam in the Skills Lab training. As with the summative examinations at the TiHo, the pass mark is 60%. Each animal clinic decides individually on the weighting of the eOSCE, although the eOSCE is administered by all TiHo clinics as a non-compensatory form of examination. This means that students must achieve the pass mark at each eOSCE station. In one clinic, the eOSCE serves as a constructive feedback tool on current knowledge and proficiency in performing practical skills. Other clinics require passing the eOSCE for certain tasks, such as participation in surgeries within the clinic rotation. If stations are not passed, the students in question appear in the CSL soon afterwards and revise the station topic with a CSL staff member. There will not be a repeat eOSCE examination. Following the revision, the students are allowed to participate in the appropriate tasks in the clinic.

The evaluation of the eOSCE is automated by the service provider usually on the day of the exam. The results of the eOSCE can be viewed by CSL staff in the examination platform. The examination results and feedback on the eOSCE are sent to the students promptly, thus avoiding delayed feedback and the associated dissatisfaction among the students (21).

### 2.8. Evaluation of the eOSCE

Within the context of this study, a questionnaire evaluating the eOSCES was distributed to PY students in the winter semester 2021/2022 and summer semester 2022 after the eOSCE. Participation was voluntary and was anonymized by ciphering. The 52 evaluation questions covered the topics of demographic data, preparation for the eOSCE, the eOSCE procedure, eOSCE structure, and feedback after the eOSCE. Students were able to rate the statements using a rating scale and voluntarily provide additional comments and observations in a free text field at the end of the questionnaire.

From May 15 to July 1, 2022, a link to an online questionnaire was sent to former and current reviewers of eOSCEs in the CSL as well as to staff members of the animal species clinics involved in the review process. The questionnaire was compiled and carried out using the online software LimeSurvey^®^ (LimeSurvey GmbH, Hamburg, Germany). Participation was voluntary and anonymised. A privacy statement had to be agreed to in advance. The questionnaire comprised a total of 97 questions and covered the following topics: demographic data, preparation for the eOSCE as an examiner, preparation for the eOSCE as an exam initiator, review process, eOSCE procedure, eOSCE structure, feedback after the eOSCE, satisfaction with the eOSCE exam format, comparison of the OSCE and eOSCE exam formats. Participants were able to rate the statements using a rating scale and answer selected questions in free text.

All questions used in the questionnaires were either validated internally by the e-learning consulting staff or have already been validated from the literature (12, 22, 23).

### 2.9. Statistical analysis

Die descriptive evaluation of the questionnaires took place with the help of the program Microsoft^®^ Office Excel 2016 (Microsoft Corporation, Redmond, WA, United States) und SAS^®^ Software, Version 9.4 and SAS^®^ Enterprise Guide^®^ 7.1 (SAS Institute Inc., Cary, NC, United States). The sample size included 175 PY-students and 27 examiners or reviewers of the eOSCEs.

### 2.10. Ethical declaration

This study was conducted in accordance with the ethical standards of the University of Veterinary Medicine Hannover, Foundation. The university’s doctoral committee, acting as the university’s ethics committee, validated the project in accordance with ethical guidelines for research with human participants and approved the study. The Data Protection Officer reviewed the project for compliance with data protection law and granted permission to conduct the study. All data obtained were processed and analyzed anonymously and in accordance with EU Regulation 2016/679 DSGVO.

## 3. Results

### 3.1. Evaluation of eOSCEs by students

**Figure 3:**
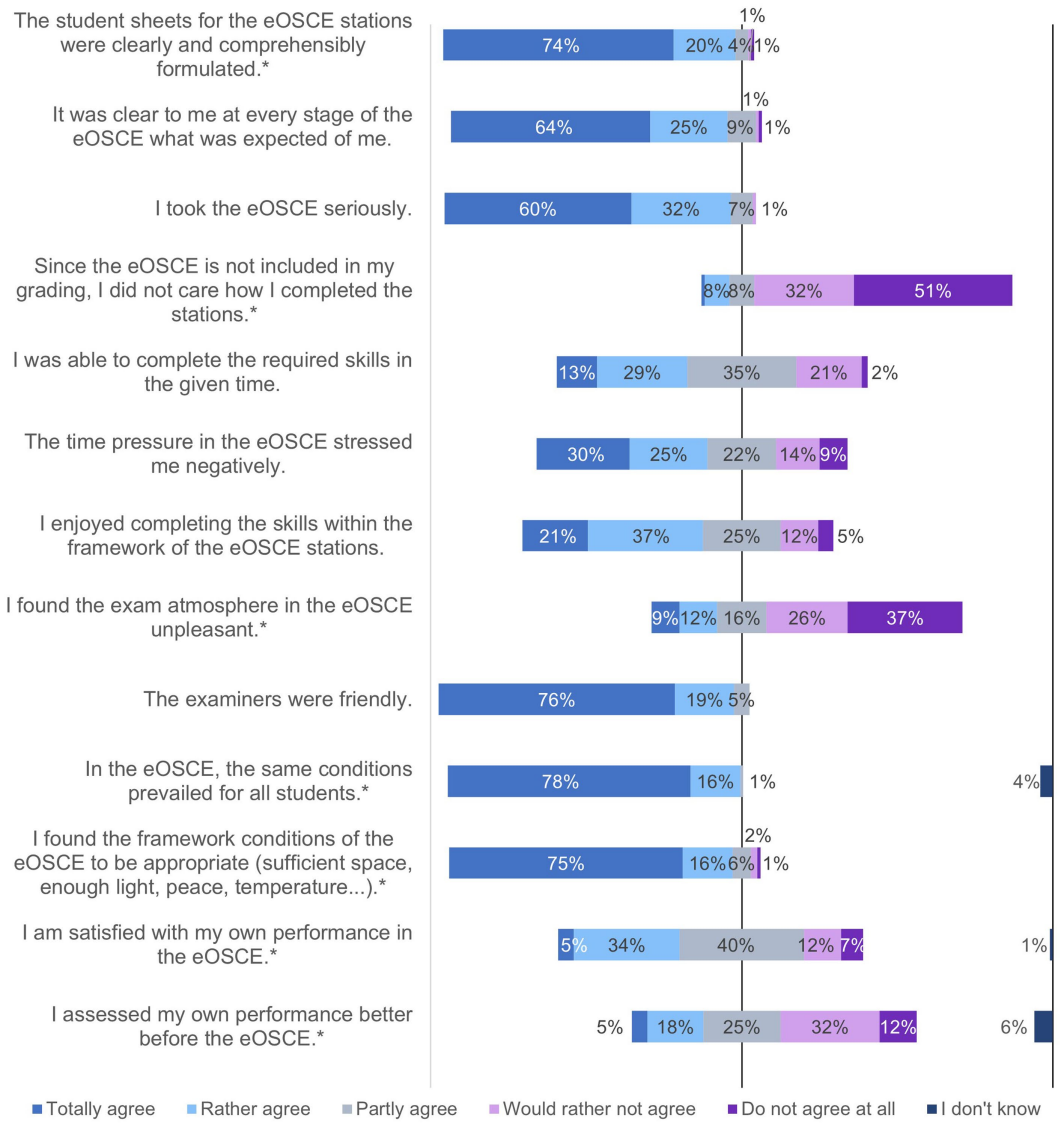
Evaluation of the eOSCE by students of the University of Veterinary Medicine Hannover in the period of September 2021 to July 2022: eOSCE procedure (*n* = 175). * = Due to correct rounding, the total is not exactly 100%.

**Figure 4:**
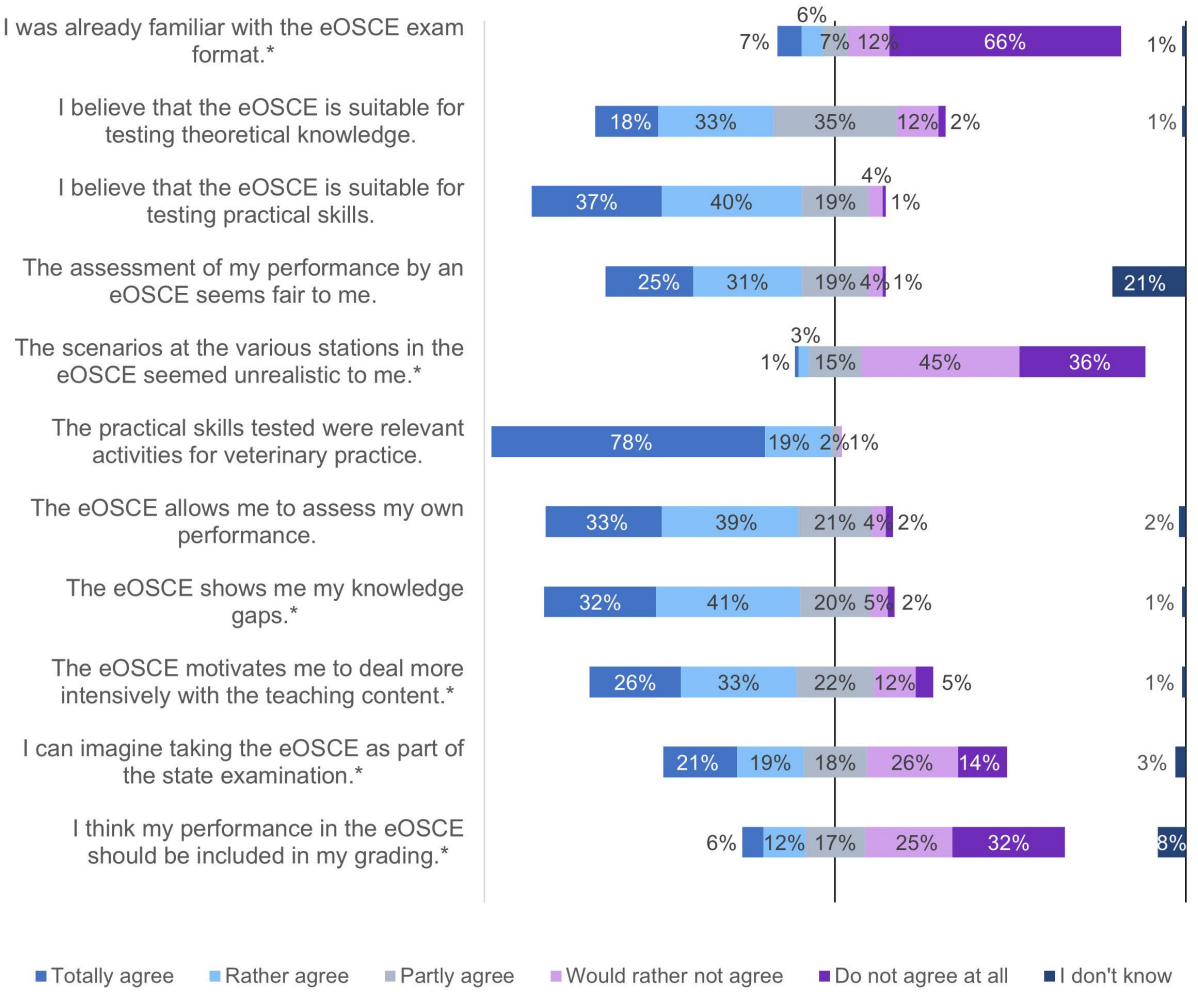
Evaluation of the eOSCE by students of the University of Veterinary Medicine Hannover in the period from September 2021 to July 2022: eOSCE structure (*n* = 175). * = Due to correct rounding, the total is not exactly 100%.

**Figure 5:**
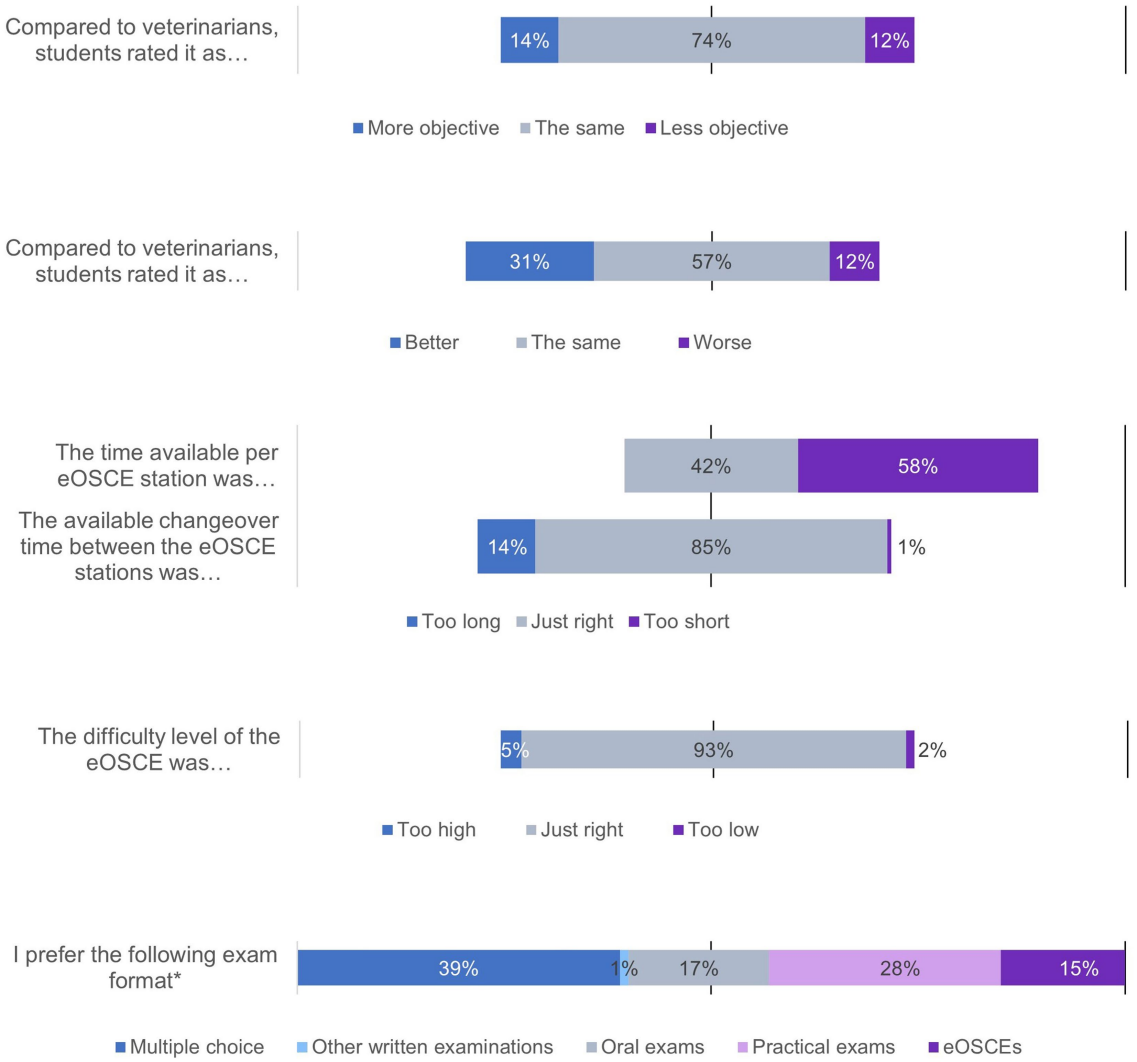
Evaluation of the eOSCE by students of the University of Veterinary Medicine in the period from September 2021 to July 2022: eOSCE structure (*n* = 175). * = Due to correct rounding, the total is not exactly 100%.

**Figure 6:**
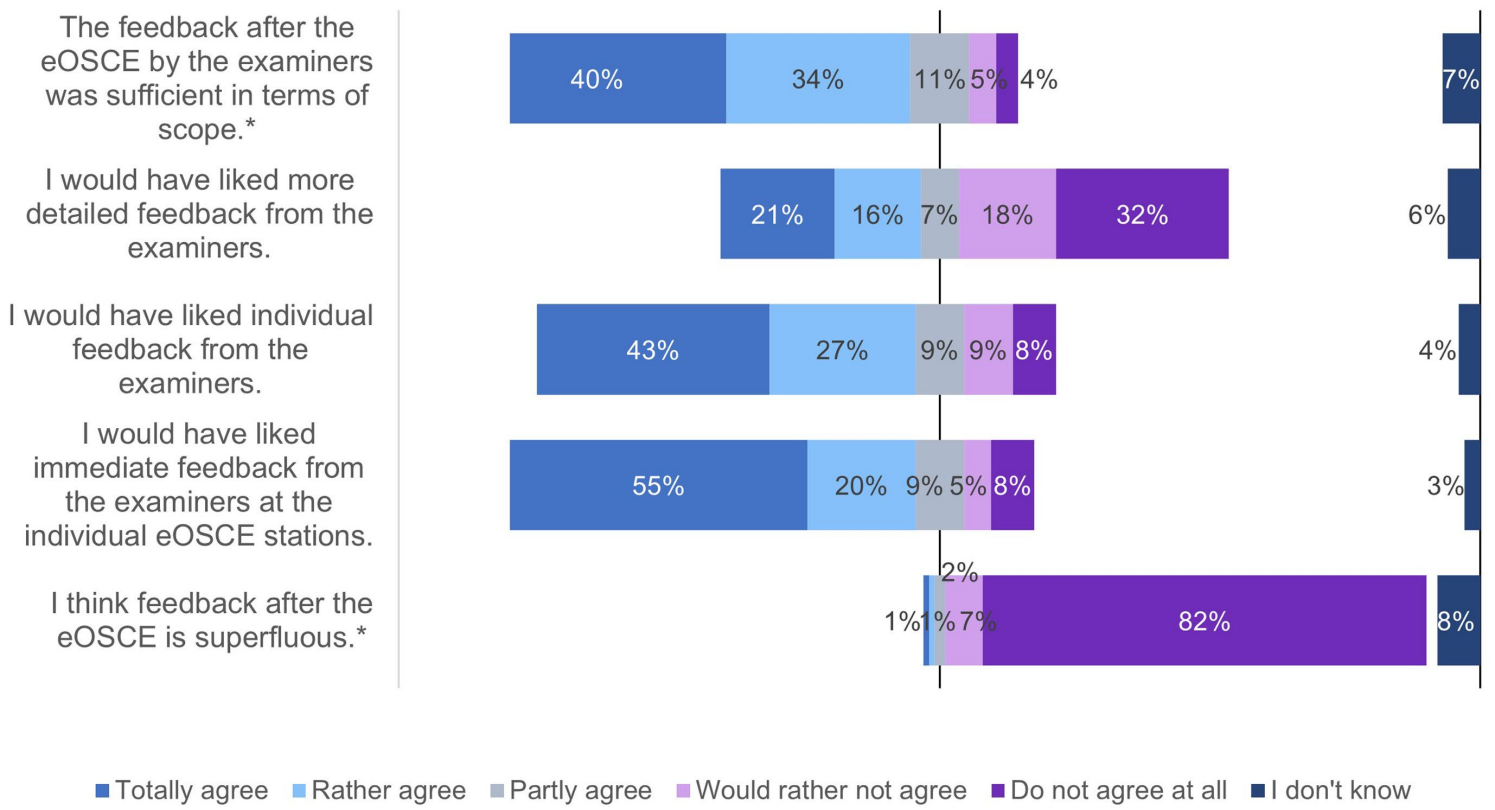
Evaluation of the eOSCE by students of the University of Veterinary Medicine Hannover in the period from September 2021 to July 2022: feedback after the eOSCE (*n* = 175). * = Due to correct rounding, the total is not exactly 100%.

The PY students of the clinics received the questionnaire for the evaluating the eOSCE each time after its completion. In this way, 175 students could be interviewed. The questions on various topics could be answered using a rating scale (Figures 1, 3–6).

In the first question block, the students answered questions about their preparation for the eOSCE (Figure 1). The majority of students felt well prepared for the eOSCE, both with the tasks in the CSL and with the materials provided. Just under a quarter of the students wanted to have more study materials to prepare themselves for the eOSCE. Half of the students fully agreed (21%) or somewhat agreed (29%) with the statement that they prepared intensively the day before the eOSCE, with over half of the students (55%) indicating a preparation time of 1–3 h.

In the second question block, the students were asked about the eOSCE process (Figure 3). More than 80% of the students stated that they took the eOSCE seriously, even though it was not summative in nature. Three quarters of the students felt at least partially negatively stressed by the time pressure. With regard to the friendliness of the examiners, the equality of the examination conditions for the students, and the spatial conditions, the reactions of the students were predominantly positive. Almost 40% of them were fully satisfied (5%) or rather satisfied (34%) with their performance in the eOSCE, and about half of the students stated that they had rated their performance at least partially better before the eOSCE.

In the third question block, students answered questions about the structure of the eOSCE (Figures 4, 5). A total of 66% of the students were not previously familiar with the eOSCE examination format, but felt that it was a fair way of assessing their performance. In this regard, 75% of students indicated that they felt the eOSCE was suitable for testing practical skills, while just under half of them felt it was also suitable for testing theoretical skills. Nonetheless, only 21% of students could imagine taking the eOSCE as a summative examination as part of the state examination.

Regarding the participation of students as examiners in the eOSCE, more than half of the students stated that they did not expect any difference in the evaluation compared to female veterinarians. The available examination time of 7 minutes was perceived as too short by 58% of the students, while they predominantly considered the given time to change examination rooms as sufficient. The difficulty level of the eOSCE was rated as just right by 93% of the students. When asked about their preferred exam format, 39% indicated multiple choice, followed by practical exams (28%) and oral exams (17%). A total of 15% of students preferred the eOSCE.

Finally, students were asked about feedback after the eOSCE (Figure 6). Almost three-quarters felt that the group feedback following the eOSCE was helpful and sufficient in terms of its scope. The majority of students would also have liked individual feedback from the examiners, especially directly after the respective eOSCE station.

### 3.2. Evaluation of the eOSCEs by the examiners

**Figure 7:**
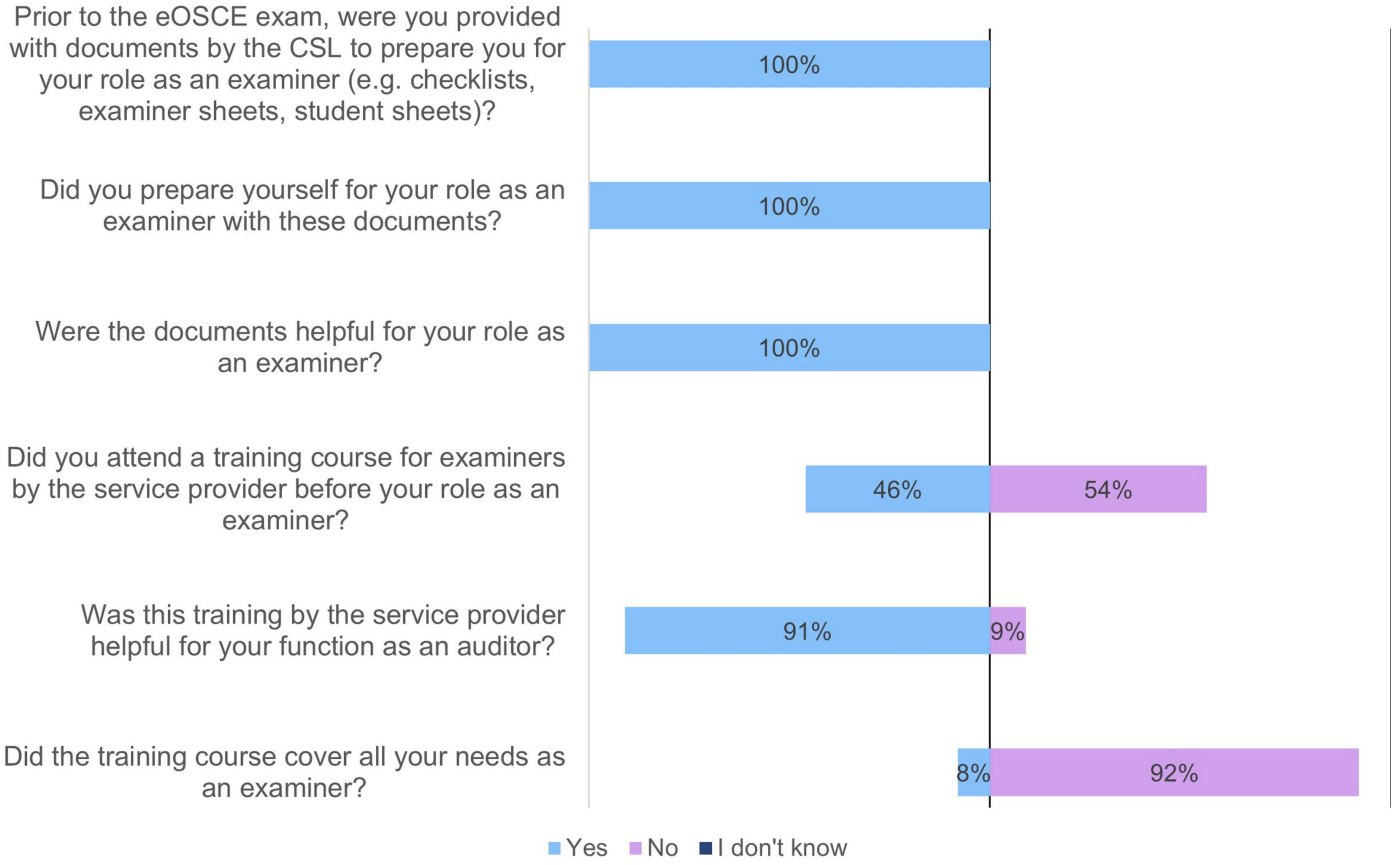
Evaluation of the eOSCE by the examiners at the University of Veterinary Medicine Hannover: preparation as examiner for the eOSCE (*n* = 24).

**Figure 8:**
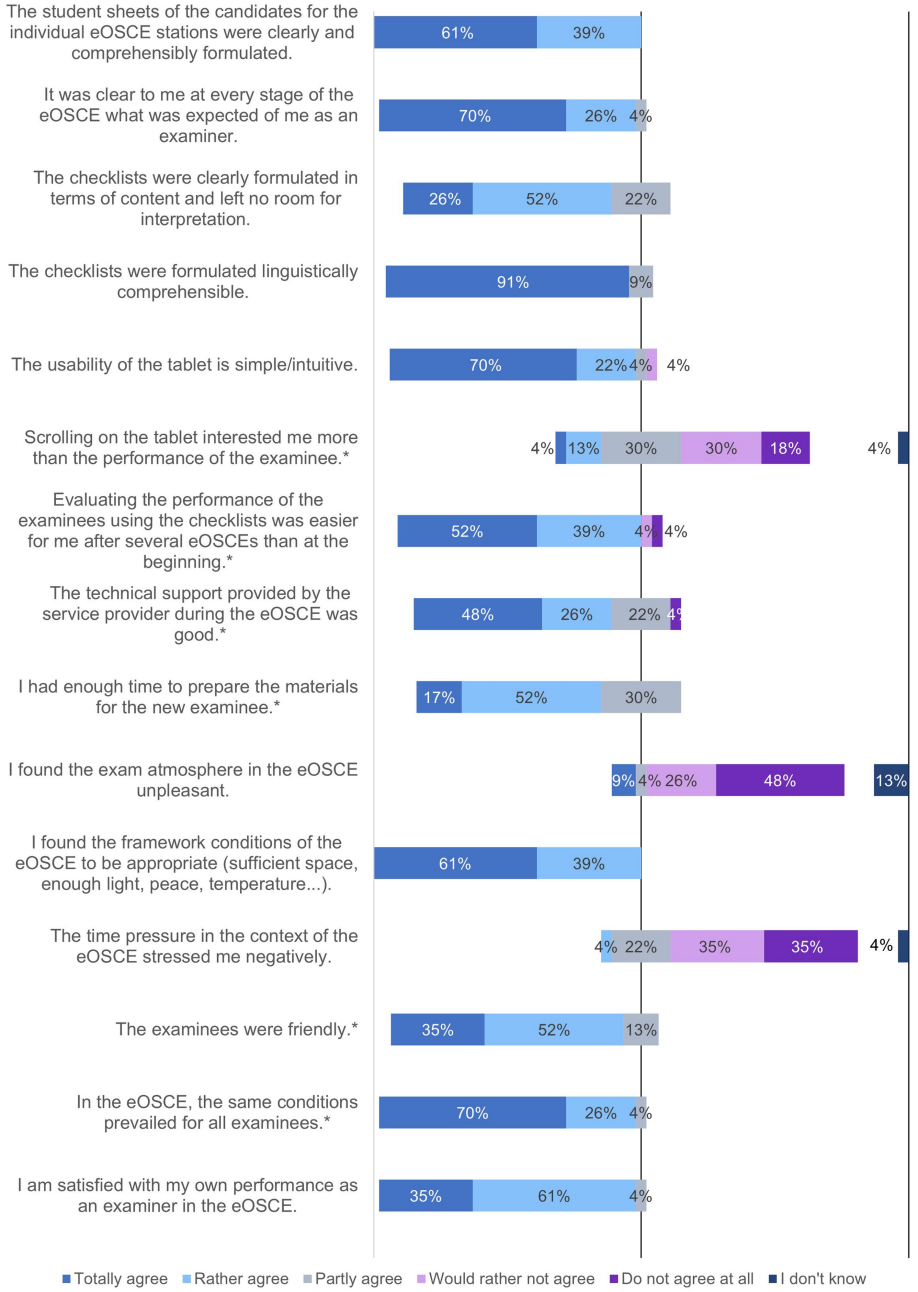
Evaluation of the eOSCE by the University of Veterinary Medicine: eOSCE procedure for examiners (*n* = 24). * = Due to correct rounding, the total is not exactly 100%.

**Figure 9:**
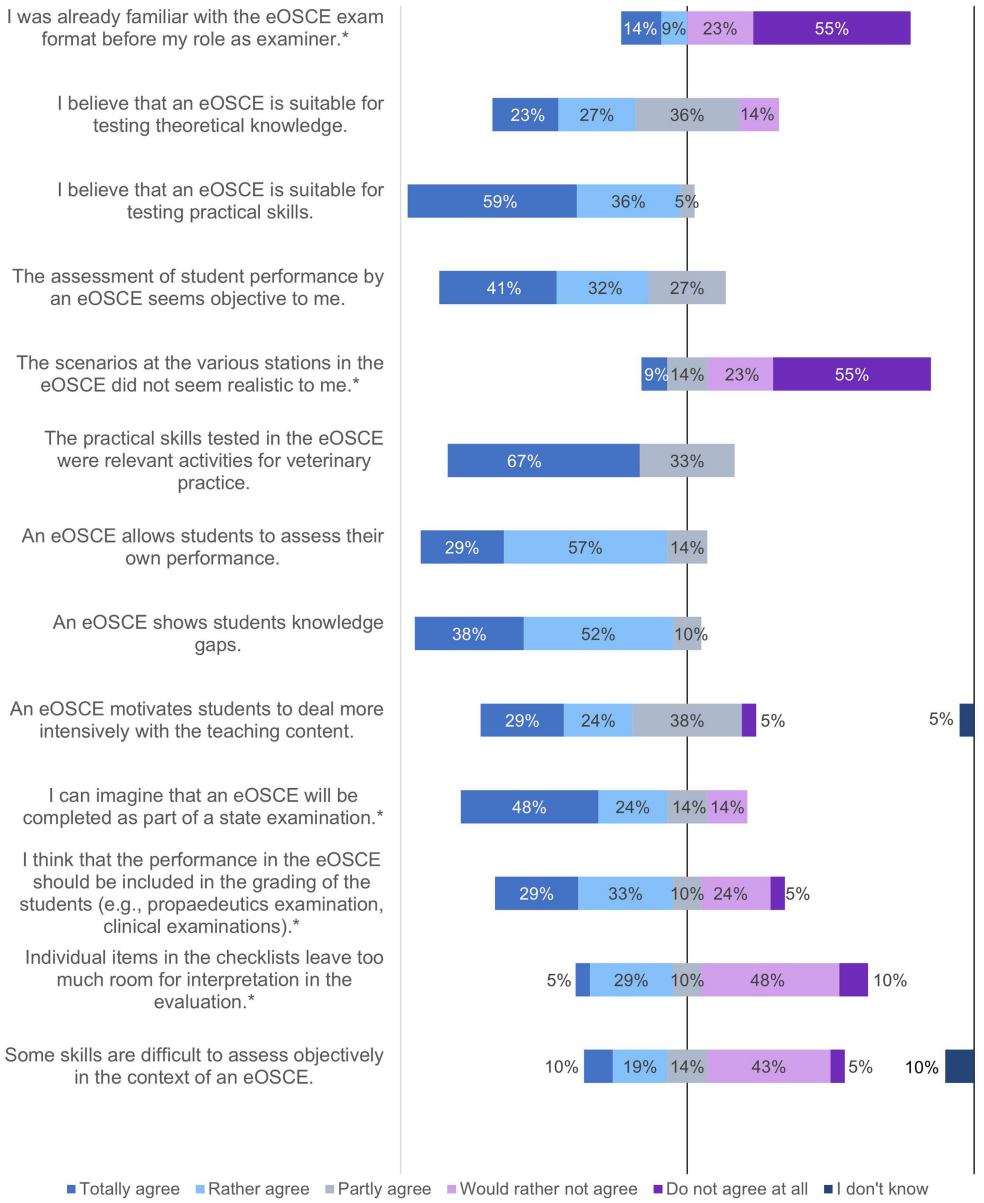
Evaluation of the eOSCE by examiners of the University of Veterinary Medicine Hannover: structure of eOSCE for examiners (*n* = 24). * = Due to correct rounding, the total is not exactly 100.

**Figure 10:**
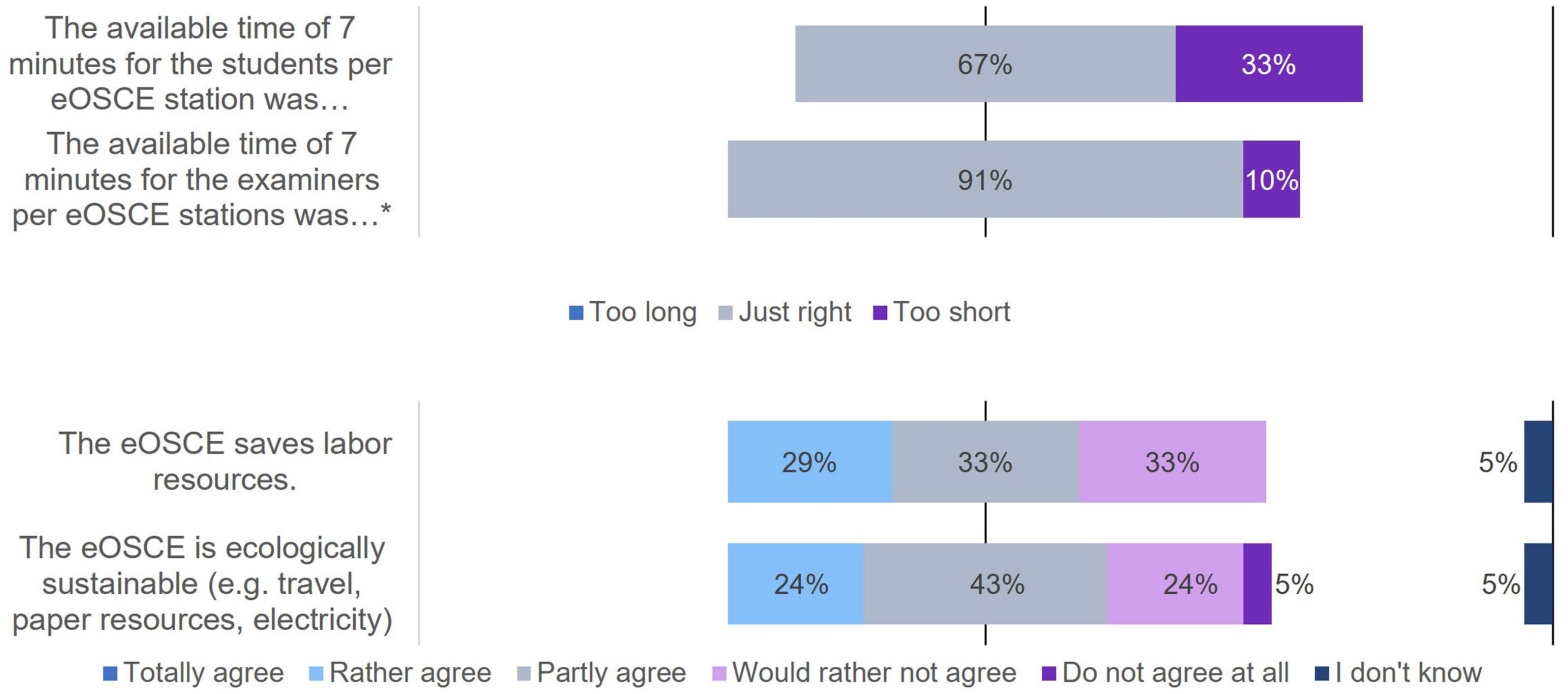
Evaluation of the eOSCE by examiners of the University of Veterinary Medicine Hannover: structure of the eOSCE for examiners (*n* = 24). * = Due to correct rounding, the total is not exactly 100%.

**Figure 11:**
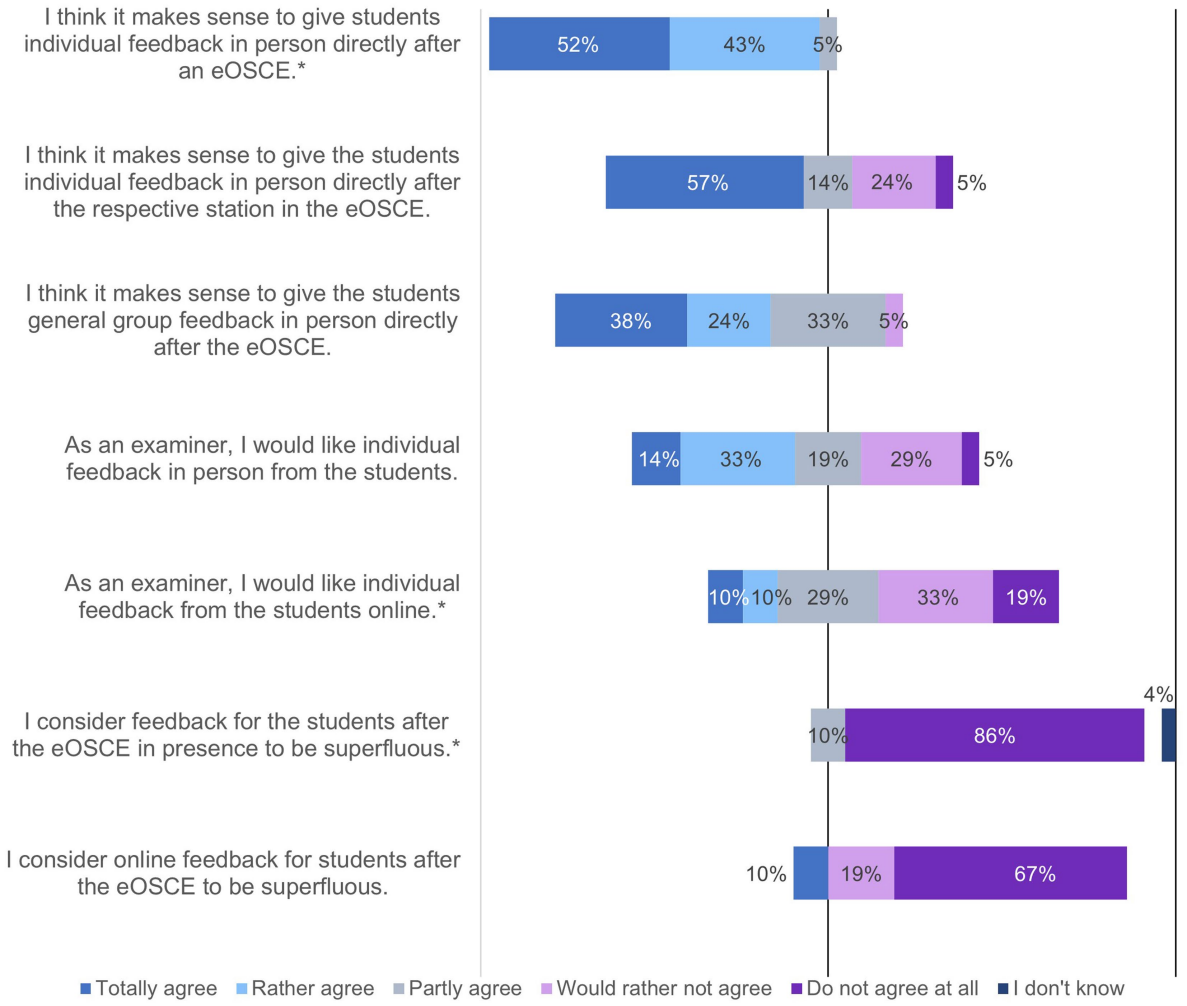
Evaluation of the eOSCE by examiners of the University of Veterinary Medicine Hannover: feedback after the eOSCE for examiners (*n* = 24). * = Due to correct rounding, the total is not exactly 100%.

**Figure 12:**
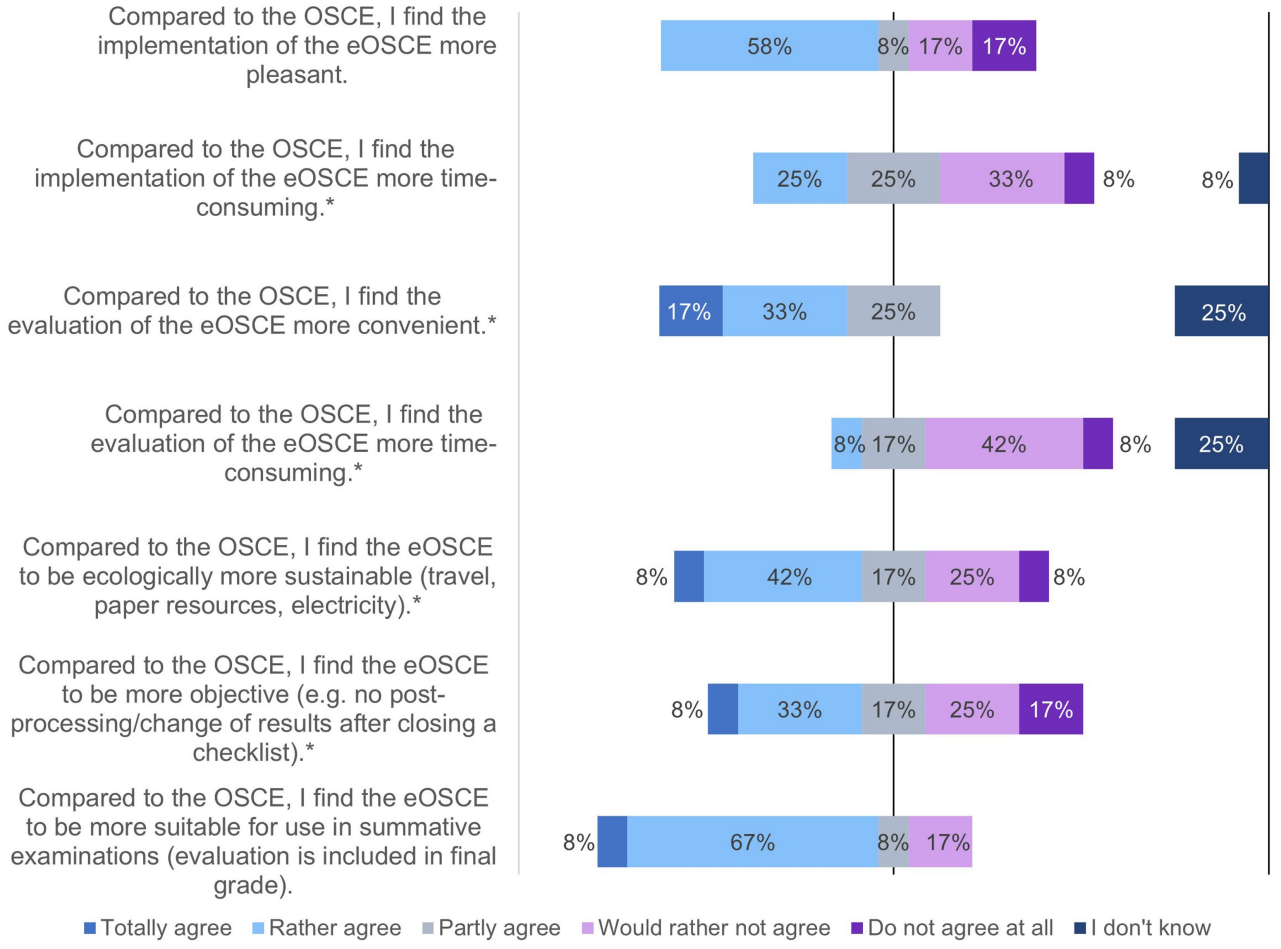
Evaluation of the eOSCE by examiners of the University of Veterinary Medicine Hannover: Comparison of OSCE with eOSCE by examiners (*n* = 12). * = Due to correct rounding, the total is not exactly 100%.

**Figure 13:**
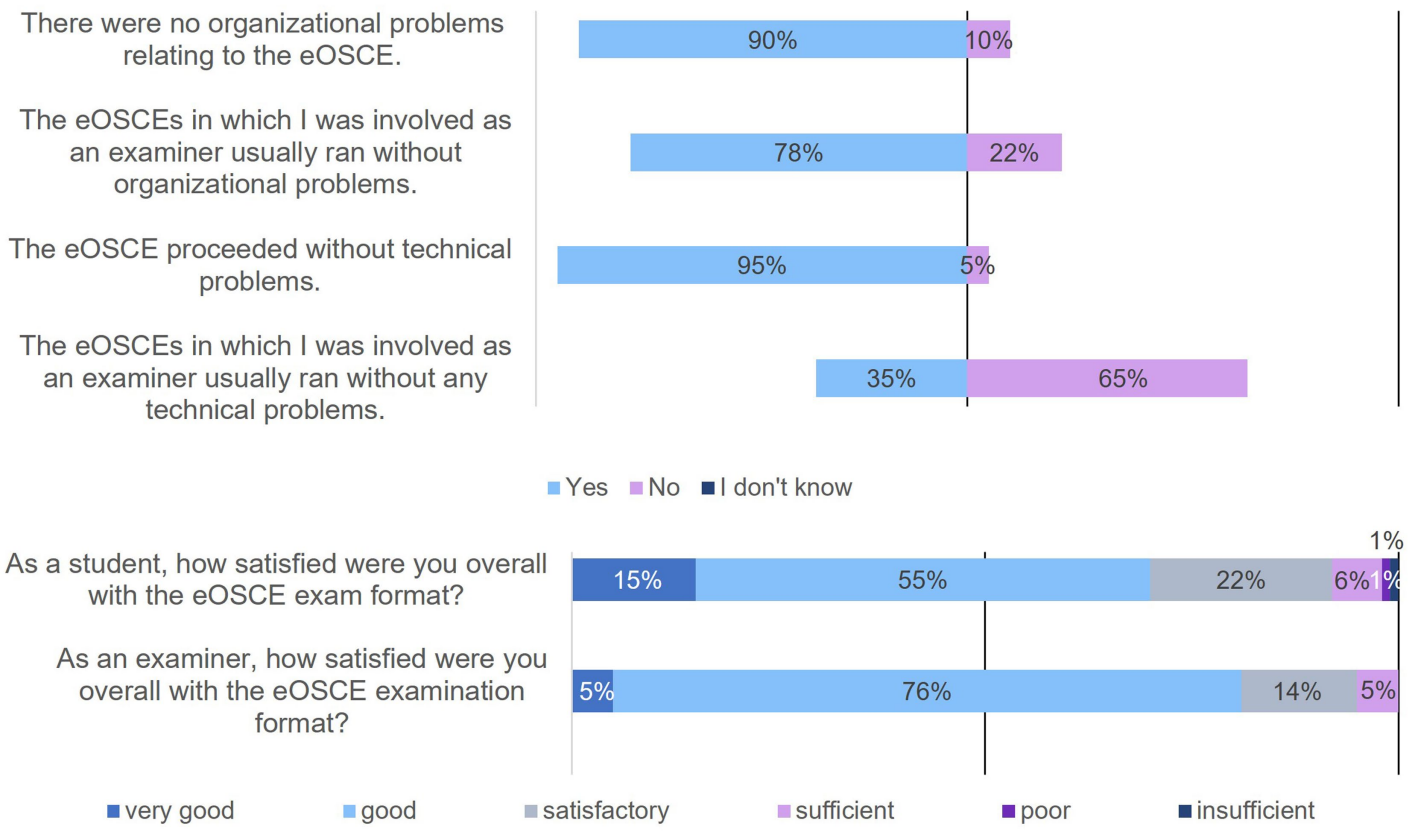
Evaluation of the eOSCE by students and examiners at the University of Veterinary Medicine Hannover: organizational and technical problems as well as student (*n* = 175) and examiner (*n* = 24) satisfaction with the eOSCE.

Between May 15 and July 1 2022, former and current examiners and reviewers were able to assess the eOSCE *via* an online questionnaire. During this period, 27 reviewers were surveyed (Figures 7–13).

In this context, 24 of the 27 participants in the survey stated that they had already completed an eOSCE as an examiner (Figure 7). All of them had prepared themselves with the documents from the CSL and found them helpful; the majority of them also found the briefing by the service provider helpful (91%).

Four survey participants had already managed at least one eOSCE as an initiator. Three of the four initiators stated that they had already experienced problems when creating one or more eOSCEs, such as problems with the checklists used and internet connection problems with the examination platform.

Seven survey participants were involved in the technical review process for one or more eOSCEs. Fifty-seven percent indicated that problems occurred during the review of at least one eOSCE, which included internal disagreements to agree on a technically correct answer as well as technical problems and difficulties understanding the exam platform. A total of 57% of reviewers found a review in the eOSCE platform more impractical than in an open format such as a Word document.

A total of 24 of the 27 participants in the survey had already used the eOSCE at least once as examiners (Figure 8). The majority found the tablet intuitive to use, but indicated that they found it easier to assess students using the checklist at the end of the eOSCE than at the beginning.

Similar to students, the majority of examiners found the eOSCE suitable for testing practical skills, and 71% could envisage the exam format as part of a state exam (Figure 9). A total of 29% of the respondents found that some skills were difficult to assess objectively in an eOSCE. Respondents cited communication with pet owners and rectal examinations as examples of these skills, citing primarily the limited exam time during an eOSCE. In addition, the transfer of the simulation to reality was criticized, since the cooperation of several persons, which is necessary in many cases for adequate patient care, was not simulated in the eOSCE.

One third of the surveyed examiners felt that the available examination time of seven minutes was too short for the students (Figure 10). A total of 29% believed that an eOSCE saved labor resources, and 24% considered the eOSCE to be environmentally sustainable in terms of travel by service provider staff and paper and electricity consumption.

The majority of examiners (95%) believed that individual feedback for students after the eOSCE was useful (Figure 11), and 57% would like to provide this individual feedback immediately following each eOSCE station. Feedback in the group following the eOSCE was also considered useful by 62% of the examiners.

Examiners who participated in both a traditional paper-based OSCE and an electronic eOSCE were asked to compare the two forms of examination (Figure 12). Of the 12 participants surveyed, 58% said they found the eOSCE more convenient and less time-consuming to conduct and also to evaluate (50%). Three quarters of the respondents considered the eOSCE to be more suitable for use in summative examinations than the OSCE.

Not only the students but also the examiners were asked about organizational and technical problems during the eOSCEs (Figure 13). A total of 10% of the students stated that they had encountered organizational problems. In the following free text field, these students could voluntarily state the type of organizational problem. These included missing material at the stations (*n* = 6 of 175), problems with the simulators (*n* = 4 of 175), and a change in the order of the stations (*n* = 2 of 175). Among the surveyed examiners, organizational problems occurred in 22% of the cases. They, too, could indicate in a subsequent free-text field what the organizational problem was. For example, incorrect sequences of students or stations (*n* = 3 of 24), missing or incorrect material (*n* = 2 of 24), and delays and insufficient assistance (*n* = 3 of 24) were mentioned. When asked if the eOSCE took place without technical problems, 95% of students answered yes, while 65% of examiners answered no. In the following voluntary free text field, examiners overwhelmingly cited problems with the tablets (*n* = 9 of 24), including tablet connection problems with the server (*n* = 4 of 24), multiple tablet freezes or crashes while completing the checklist (*n* = 9 of 24), and no automatic opening of the checklist at the beginning of the exam (*n* = 2 of 24).

Additionally, students and examiners were asked about their overall satisfaction with the eOSCE format (Figure 13) and were then able to provide additional comments in free text. Students gave the eOSCE an average rating of 2.3, while examiners gave an average rating of 2.2. In the free text, students particularly wanted more practice time (*n* = 20 of 175) in the preceding skills lab training, and personal individual feedback from the examiners in the respective eOSCE station (*n* = 16 of 175). Examiners questioned the usefulness of the eOSCE in terms of environmental and economic sustainability, especially against the background of very small examination groups of 1–6 persons (*n* = 3 of 24). They also wanted direct problem solving by telephone from the support team (*n* = 3 of 24), faster resolving of problems that arise (n = 3 of 24), and optimization of the handling of the checklists on the tablet (*n* = 3 of 24). A suggestion from the examiners was also to offer the possibility in future to give students individual feedback directly after the respective eOSCE station (*n* = 2 of 24).

## 4. Discussion

The results of the study show that both students and examiners were satisfied with the eOSCE overall. Students and examiners considered the eOSCE to be suitable for better assessing student performance and identifying knowledge gaps. Although in this study Examiners compared traditional paper-based OSCE and electronic eOSCE (Figure 12), the evaluation of the eOSCE was found without a direct comparison to a paper-based OSCEs The positive impact of the eOSCE on student learning and the curriculum was already demonstrated in several studies (24–26) and is an important feature of examinations in terms of the so-called educational impact (23, 27). In this context, feedback for students also plays a major role. In the questionnaire, students and examiners were in favor of individual feedback, preferably directly following the respective eOSCE station. Allen et al. (28) already showed that a one and a half minute feedback after the OSCE station is perceived by students as helpful and not disturbing and also leads to an improvement in student performance (29). On the other hand, it may be difficult to integrate detailed feedback into a summative OSCE, especially if the individual stations are thematically similar, and thus some students may gain an advantage for the following station by receiving direct feedback. In principle, students’ experiences during the eOSCE can help optimize further learning even without any direct feedback (15).

The majority of students believe that student examiners assess their performance as well and objectively as veterinarians. Numerous studies in the field of human medicine can be found in the literature, which come to different conclusions (12, 30–36). Some studies found good agreement in examiner quality when comparing student examiners and physicians (12, 30–32), while other studies concluded that students are only partially suitable or not suitable as examiners (33, 34). It was found to be problematic that students have inhibitions about evaluating each other poorly (35) and are themselves of the opinion that they cannot objectively evaluate the performance of their fellow students (36). Basically, students from higher semesters seem to be able to act as examiners in OSCEs of lower semesters if they are well trained and if they are able to assess their fellow students objectively (12, 32, 34). In the present study, the majority of examiners consisting of veterinarians and students expressed satisfaction with their examination performance in the eOSCE. Some examiners indicated that they found it difficult to assess skills such as owner communication with a checklist.

Newble (18) recommends the use of a global rating for OSCE stations centered to assess communications skills. This can better test competencies in the areas of empathy and ethics (37, 38) and leads to increased use of open-ended questions by students at the OSCE station (39). Alternatively, a combination of a classic checklist and global rating is also possible (40) When comparing the OSCE with the eOSCE, no clear finding could be obtained in the present study. In contrast, other studies already showed that examiners prefer electronic systems to paper for reviewing students (41–43). The eOSCE was also convincing in other studies with regard to the higher reliability of checklist completion by examiners (44), saving time in the area of planning and evaluation (41, 45), and the rapid sending of examination results to students (45), especially for large examination groups (46, 47). A tendency in this direction is also evident in this study, especially with regard to the use of the eOSCE as a summative examination. The time saved in creating and evaluating the eOSCE also seems to be a clear plus point for many participants in this study.

In conclusion, it can be said that the examination format eOSCE is well accepted as a feedback instrument by both students and examiners at TiHo. With regard to the use of the eOSCE as a summative examination in the state examination, the group of students in particular had diverging opinions. In order to achieve successful summative eOSCEs, the provided software has to be stable and reliable. When deciding whether to use an electronic OSCE instead of a paper-based OSCE, the cost–benefit ratio and different systems should be examined in advance. An added value of eOSCEs is the fast and reliable evaluation of students’ OSCE performance as well as the digital recording of exam results. The exam time should be well calculated so as not to unnecessarily increase the stress of the students due to too little time.

## Data availability statement

The raw data supporting the conclusions of this article will be made available by the authors, without undue reservation.

## Author contributions

SB and SW conceived and designed the study. SW, AT and ES supervised the study. SB, SW, and AT created the questionnaire for the evaluation. SB collected, analyzed, and interpreted the data, which was supervised by SW. The manuscript was drafted by SB and all authors reviewed and edited the manuscript. All authors contributed to the article and approved the submitted version.

## Funding

This Open Access publication was funded by the Deutsche Forschungsgemeinschaft (DFG, German Research Foundation) - 491094227 Open Access Publication Funding and the University of Veterinary Medicine Hannover, Foundation.

## Conflict of interest

The authors declare that the research was conducted in the absence of any commercial or financial relationships that could be construed as a potential conflict of interest.

## Publisher’s note

All claims expressed in this article are solely those of the authors and do not necessarily represent those of their affiliated organizations, or those of the publisher, the editors and the reviewers. Any product that may be evaluated in this article, or claim that may be made by its manufacturer, is not guaranteed or endorsed by the publisher.
